# Flux balance analysis reveals acetate metabolism modulates cyclic electron flow and alternative glycolytic pathways in *Chlamydomonas reinhardtii*

**DOI:** 10.3389/fpls.2015.00474

**Published:** 2015-06-30

**Authors:** Stephen P. Chapman, Caroline M. Paget, Giles N. Johnson, Jean-Marc Schwartz

**Affiliations:** Faculty of Life Sciences, University of ManchesterManchester, UK

**Keywords:** glycolysis, mixotrophic growth, metabolic model, photosynthesis, green algae, flux balance analysis, acetate metabolism, cyclic electron flow

## Abstract

Cells of the green alga *Chlamydomonas reinhardtii* cultured in the presence of acetate perform mixotrophic growth, involving both photosynthesis and organic carbon assimilation. Under such conditions, cells exhibit a reduced capacity for photosynthesis but a higher growth rate, compared to phototrophic cultures. Better understanding of the down regulation of photosynthesis would enable more efficient conversion of carbon into valuable products like biofuels. In this study, Flux Balance Analysis (FBA) and Flux Variability Analysis (FVA) have been used with a genome scale model of *C. reinhardtii* to examine changes in intracellular flux distribution in order to explain their changing physiology. Additionally, a reaction essentiality analysis was performed to identify which reaction subsets are essential for a given growth condition. Our results suggest that exogenous acetate feeds into a modified tricarboxylic acid (TCA) cycle, which bypasses the CO_2_ evolution steps, explaining increases in biomass, consistent with experimental data. In addition, reactions of the oxidative pentose phosphate and glycolysis pathways, inactive under phototrophic conditions, show substantial flux under mixotrophic conditions. Importantly, acetate addition leads to an increased flux through cyclic electron flow (CEF), but results in a repression of CO_2_ fixation via Rubisco, explaining the down regulation of photosynthesis. However, although CEF enhances growth on acetate, it is not essential—impairment of CEF results in alternative metabolic pathways being increased. We have demonstrated how the reactions of photosynthesis interconnect with carbon metabolism on a global scale, and how systems approaches play a viable tool in understanding complex relationships at the scale of the organism.

## Introduction

Photosynthesis uses light energy to fix atmospheric CO_2_ into organic molecules, which are incorporated into carbon skeletons and can be used to produce biomass or broken down to provide ATP and reducing power. *Chlamydomonas reinhardtii* has been widely used as a model organism to study photosynthesis (Harris, 2001) but more recently has received attention as a potential system for producing biofuels (Hannon et al., 2010). *C. reinhardtii* is a facultative phototrophic organism; it can use light and atmospheric CO_2_ as the sole carbon source but can also grow mixotrophically when supplemented with additional inorganic carbon, such as acetate. Mixotrophic growth typically results in an enhanced biomass, however at a cost of a reduced photosynthetic capacity (Johnson and Alric, 2012; Plancke et al., 2014).

It has been widely reported that providing cells with acetate results in an inhibition of photosynthesis (Johnson and Alric, 2013; Johnson et al., 2014; Plancke et al., 2014). Assimilation of acetate might occur via either the glyoxylate or the tricarboxylic acid (TCA) cycle, providing carbon skeletons and reducing equivalents to drive metabolism (Roach et al., 2013). A key requirement of this suggested assimilation is a source of ATP since the incorporation of acetate into metabolism can occur along one of two pathways, both requiring ATP: a direct conversion with acetyl CoA synthase (ACS) or a pathway involving acetate kinase (ACK) and phosphate acetyltransferase (PAT) (Johnson and Alric, 2013). The optimal functioning of photosynthesis depends on there being a balance in the stoichiometry of the products of electron transport, namely NAPDH and ATP. Assimilation of acetate is liable to disturb this balance, consuming ATP and producing reducing equivalents. It has been suggested that this imbalance results in a re-routing of the photosynthetic electron transport chain, from linear flow, involving both Photosystem (PS) II and PSI, to a cyclic flow, only involving PSI. This cycling of electrons around PSI results in the generation of ATP without producing NADPH, giving rise to a decrease in oxygen evolution (Munekage et al., 2008). To gain a further understanding of this photosynthetic acclimation response to acetate, we need to investigate how acetate is integrated into metabolism at an organismal level. The availability of a full genome sequence for this species (Merchant et al., 2007) means that we can now adopt a systems level approach to understanding how acetate modulates metabolism. All known metabolic pathways encoded by the genome of *C. reinhardtii* have been mapped in a global representation of its metabolism. With this knowledge, several methods for analysing metabolism have been developed.

One of the most popular methods for analysing metabolic pathways is constraint based modeling, which includes the techniques of Flux Balance Analysis (FBA) and Flux Variability Analysis (FVA) for the prediction of metabolite flux through a metabolic network reconstruction (Orth et al., 2010). Flux based modeling allows for the inclusion of realistic constraints imposed upon the system, for example maximal nutrient uptake/excretion rates. As a result, growth rates and production of biotechnologically important metabolites can be predicted using constraint based methods in conjunction with genome-scale models, which contain all known metabolic information (Schilling et al., 1999). FBA was first used by Boyle and Morgan (2009) to address carbon metabolism in a small scale model of *C. reinhardtii*. More recently, two genome scale reconstructions (GEM) of this organism were presented and used for prediction of metabolic fluxes in different growth conditions (Chang et al., 2011; De Oliveira Dal'Molin et al., 2011).

The AlgaGEM model, which was derived from a previous model of *Arabidopsis thaliana* (De Oliveira Dal'Molin et al., 2010), showed substantial evidence for the accurate prediction of hydrogen production during a heterotrophic growth regime. They analyzed the competition between hydrogen production and the photosynthetic pathway of cyclic electron flow (CEF), thereby showing the importance of optimizing photosynthetic regulation for biofuel production. *i*CR1080, the other published and validated GEM of *C. reinhardtii*, was assembled following a bottom-up approach and specifically validated by examining the effects of different light spectral ranges and intensities on metabolic and photosynthetic reactions (Chang et al., 2011). The centrality of the chloroplast and the complexity of photosynthetic reactions make this model optimal for the prediction of biomass and light usage under phototrophic and mixotrophic growth conditions. In addition, starch and other carbon sinks can accumulate as biomass within *i*CR1080, giving indicative *in silico* results that can explain the photosynthetic responses to acetate. For these reasons, the *i*CR1080 model was chosen for our study.

The aim of this work was to examine how metabolic fluxes change in response to acetate addition. We have investigated the functioning and regulation of photosynthesis in *C. reinhardtii in silico* to assess photosynthetic down regulation using flux modeling techniques and validated against experimental evidence. FBA and FVA were then used to calculate flux distribution for both phototrophic and mixotrophic growth. Furthermore, a reaction essentiality analysis was performed to investigate which reactions are essential to explain observed traits. We show that the ATP requirement for acetate assimilation is indeed obtained by a re-routing of electrons around PSI, consistent with qualitative experimental published data (Munekage et al., 2008; Johnson and Alric, 2013) and an increased activity of central carbon metabolic pathways is associated to mixotrophic growth on acetate.

## Materials and methods

### Model curation

As the photosynthetic reactions are of particular importance, all photosynthetic reactions in the model were validated and corrected where necessary. Specifically, we corrected the CEF reactions, in which flux toward plastocyanin was re-directed toward cytochrome *b*_6_f, in line with published data (Alric, 2014). In addition to this, we included the movement of protons from the stroma to the lumen that occurs as a result of CEF (Johnson, 2011), which was otherwise missing in the original model (for complete SBML model, see Supplementary file S1).

### Flux balance analysis

FBA was performed using a pre-existing model of *C. reinhardtii* metabolism, *i*CR1080 (Chang et al., 2011). Maximization of biomass (μ) was used as the objective function for FBA. Flux predictions were compared between acetate-fed conditions and cells grown phototrophically. For phototrophic growth, the model inputs were represented by experimental nutrients and light uptake (constraints found in Supplementary files S2, S3) as a base model and the addition of acetate to this model represented mixotrophic growth. Additional constraints imposed on the system included constraining the dry weight of an actively metabolizing algal cell (Mitchell et al., 1992), known starch degradation rates in the light (Levi and Gibbs, 1984) and the light induced inhibition of the enzymes protochlorophyllide reductase (Cahoon and Timko, 2000), phosphofructokinase, glucose-6-phosphate-1-dehydrogenase, glucose 6-phosphate dehydrogenase (Mustroph et al., 2013), and fructose-bisphosphate aldolase (Murakami et al., 2005). The resulting fluxes in the two conditions were scored and ranked in decreasing order, based on their absolute difference.

### Flux variability analysis

FVA is an FBA-based method for characterizing all feasible states of genome-scale metabolic models that satisfy a set of given constraints and objective function (Mahadevan and Schilling, 2003). Many different combinations of flux vectors can satisfy the given objective function by using different pathways, resulting in various flux distributions. As such, the points in the solution space that maximize or minimize a given objective function can be characterized (Orth et al., 2010). Minimal and maximal possible fluxes were obtained for each reaction whilst constraining the biomass production in the model to a growth rate obtained from FBA (flux values from FVA are contained in Supplementary files S4, S5). For each reaction, the minimal possible flux under mixotrophic conditions (*a*) were subtracted from maximal fluxes under phototrophic conditions (*b*). Conversely, minimal fluxes under phototrophic conditions (*c*) were subtracted from maximal fluxes of mixotrophic conditions (*d*).

$$\begin{array}{l} a-b=x\\ c-d=y \end{array}$$

Absolute differences from the above two subtractions were calculated to give the smallest possible difference between conditions for a given reaction to satisfy the objective function of maximizing biomass in each case.

abs diff=|x−y|

For reactions where there was a clear separation between fluxes of phototrophic and mixotrophic conditions, these were classified as non-overlapping fluxes, in other words, there was always a difference in the flux between the two growth conditions. Meanwhile, overlapping fluxes were given a score of 0 and discounted. From the score established, all positive fluxes were identified. FBA and FVA were performed using the COBRA toolbox within the MATLAB (R2013a) environment, used in conjunction with Gurobi optimizer solver (version 5.6) employing a linear programming based optimization algorithm (see Supplementary file S6 for flux values obtained under phototrophic and mixotrophic conditions).

### Reaction essentiality analysis

To determine which reactions and metabolic pathways are active or dormant in a particular condition, a reaction essentiality analysis was performed from the non-overlapping reactions derived from FVA (Henry et al., 2006). For a given condition, reactions of *iCR*1080 were classified as flux essential (requiring a non-zero flux), flux substitutable (where the range of possible fluxes span zero) and flux blocked (reactions with minimum and maximum flux of zero). This method uses FVA results to determine whether a flux is always required for optimal biomass production, is potentially used for optimal biomass production or is never used for optimal biomass production.

### Cell cultivation and growth

Cultures of *C. reinhardtii* wild-type (137C) were used in this study. Cells were cultured in 250 ml conical flasks with 200 ml medium, which were placed in a shaker at 180 rpm and illuminated on a 16 h light/8 h dark cycle with light provided by warm-white LED lamps (color temperature 3000–3200 K), at 140 μmol m^−2^ s^−1^, 22°/16°C day/night. Mixotrophic cultures were grown in a Tris-Acetate-Phosphate (TAP), with added potassium phosphate (pH 7.0) and Hutner trace elements (Hutner et al., 1950) whilst phototrophic cultures were grown in light using a modified minimal medium, which TAP medium without addition of acetate. The pH of minimal and TAP media was adjusted to 7.8.

#### Cell growth analysis

For inoculation, 250 μl phototrophic cultures in exponential growth phase were axenically transferred to 250 ml conical flasks containing 200 ml of the appropriate medium. Following introduction of algae, cell growth was monitored at intervals for 12 days, until a stationary or death phase was reached. The optical density of cultures at 680 nm (OD_680_) was measured using an Ocean Optics USB4000 spectrophotometer (Ocean Optics, Dunedin, FL, USA). Specific growth rates (μ) were derived from cells in an exponential phase of growth, from two time points (*t_1_, t_2_*) with associated OD readings used in the equation below.

$$\mu =\frac{lnOD_{2}-lnOD_{1}}{\left(t_{2}-t_{1}\right)}$$

#### Chlorophyll analysis

Chlorophyll concentration was used as a variable to calculate oxygen evolution. 1.5 ml of cell suspension was centrifuged for 5 min at 2400× g. The cell pellet was re-suspended in 1.5 ml of 80% v/v acetone and homogenized vigorously. The cell samples were again centrifuged before estimating the chlorophyll content, using the method of Porra et al. (1989).

#### Oxygen evolution

Measurements of O_2_ evolution was under saturating light conditions were performed using a Hansatech oxygen electrode in a DW2 liquid-phase oxygen electrode chamber (Hansatech Instruments, Norfolk, UK). Intact *C. reinhardtii* cells in culture medium were placed into the chamber, maintained at 20°C and continuously stirred. Saturating light was provided by a Led Engin LZ4 warm white LED (LED Engin, San Jose, CA, USA) driven by a laboratory built constant current power supply. Rates of photosynthesis were normalized to cell number for each sample. Oxygen evolution was determined every 24 h from Day 4 until cells reached a stationary phase, expressed as oxygen evolution on a cellular basis.

## Results

### Simulations describe acetate assimilation into the TCA cycle

**Figure 1:**
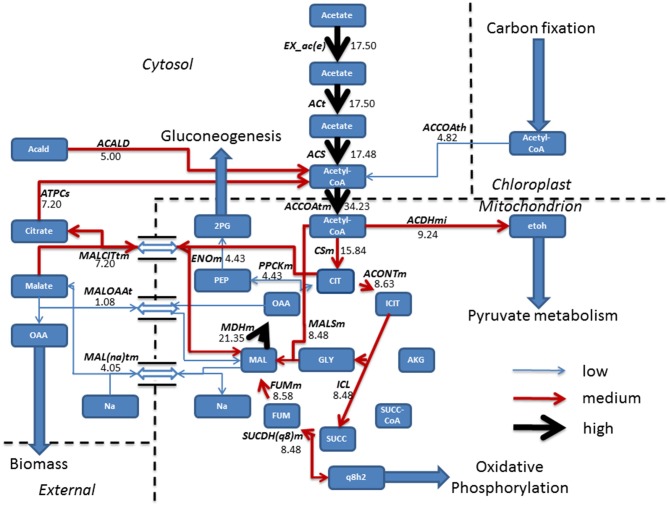
**Resulting fluxes (mmol gdw^−1^ h^−1^) from mixotrophic growth reveal the incorporation of acetate into the TCA cycle, albeit a “half TCA” cycle due to flux being carried by isocitrate lyase bypassing the CO_2_ evolving steps**. To sustain the TCA cycle, mixotrophic cells have a constant shuttle of malate from the mitochondrion to allow sodium ion, oxaloacetate import and export of citrate, used to fuel acetyl-CoA production which is further fed back into the TCA cycle or re-allocated for pyruvate metabolism. Following export of malate from the TCA cycle, conversion into oxaloacetate ensures a gain in biomass, whilst the gluconeogenesis precursor phosphoenolpyruvate is synthesized from oxaloacetate, obtained from the TCA cycle. Fluxes were categorized as low (<5 mmol gdw^−1^ h^−1^, thin blue arrow), medium, (>5–10 mmol gdw^−1^ h^−1^, red arrow), and high (>10 mmol gdw^−1^ h^−1^, thickest black arrows). Antiport reactions have been denoted by a reversible arrow. 2PG, Glycerate 2-phosphate; Acald, Acetaldehyde; ACALD, Acetaldehyde dehydrogenase (Cytosolic); ACCOAth, Acetyl-CoA/CoA antiporter; ACCOAtm, Acetyl-CoA transport; ACDHmi, Acetaldehyde-CoA dehydrogenase (mitochondrial); ACONTm, Aconitate hydratase; ACS, acetyl-CoA synthetase; ACt, acetate transport; AKG, Alpha Ketogluterate; ATPCs, ATP citrate synthase; CIT, Citrate CSm, Citrate synthase; ENOm, Enolase; etoh, Ethanol; EX_ac(e), Acetate exchange; FUM, Fumerate; FUMm, fumarate hydratase; GLY, Glyoxylate; ICIT, Isocitrate; ICL, Isocitrate lyase; Mal, Malate; MALCITtm, Malate/citrate antiporter; MAL(na)tm, Malate/sodium antiporter; MALOAAt, Malate/Oxaloacetate antiporter; MALSm, malate synthase; MDHm, malate dehydrogenase; OAA, Oxaloacetate; PEP, Phosphoenolpyruvate; PPCKm, Phosphoenolpyruvate Carboxykinase; q8h2, Ubiquinol; SUCC, Succinate, SUCC-COA; SUCDH(q8)m, Succinate Dehydrogenase.

**Figure 2:**
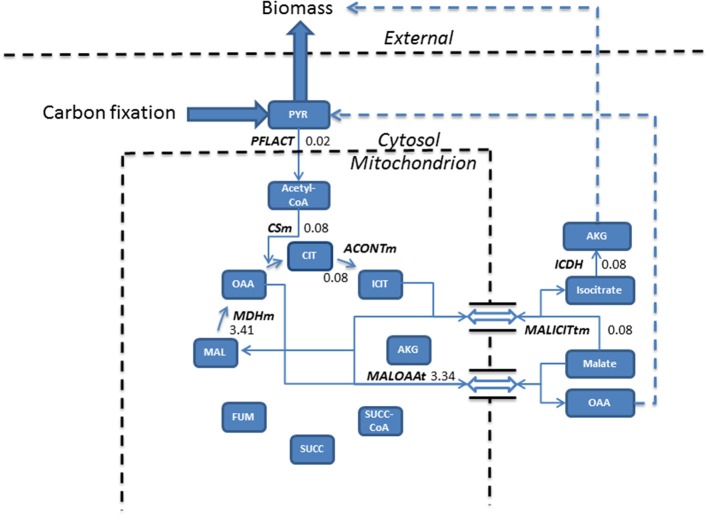
**Phototrophic simulations indicate the TCA cycle carried a much reduced flux (mmol gdw^−1^ h^−1^) than that obtained from mixotrophic simulations, resulting from the conversion of pyruvate into acetyl-CoA into the cycle**. The import of malate, exchanged for isocitrate export forms the basis of biomass production, meanwhile, malate re-entry into the cycle occurs to allow the export of oxaloacetate feeding back into pyruvate metabolism. Dashed lines assemble multiple steps for clarity. Double arrow represents antiporter reactions. AKG, alpha ketogluterate; ACONTm, Aconitate hydratase; CIT, Citrate; CSm, Citrate synthase; ICDH, Isocitrate dehydrogenase; ICIT, Isocitrate; FUM, Fumerate; MAL, Malate; MALICITtm, Malate/isocitrate antiporter; MALOAAt, Malate/Oxaloacetate antiporter; MDHm, malate dehydrogenase; OAA, Oxaloacetate; PFLACT, Formate C-acetyltransferase; PYR, Pyruvate; SUCC, Succinate; SUCC-CoA, Succinyl-CoA.

FBA was employed for the prediction of metabolic fluxes that vary as a result of acetate introduction, giving a theoretical description of pathways that are altered following addition of acetate to induce mixotrophic growth. During phototrophic growth, the cell fixed carbon dioxide by converting light into cellular energy (reducing equivalents and ATP) whilst mixotrophic growth was simulated using the phototrophic model as a base model and allowing the uptake of acetate at an experimentally defined rate (Hoober, 1989). All other constraints imposed on the system were conserved. Steady state metabolic flux distribution was mapped for both phototrophic and mixotrophic growth. Under mixotrophic conditions, the largest subset of reactions showing the greatest change in flux involved acetate assimilation and integration into the TCA cycle (Figure 1). This carried flux an order of magnitude higher than in phototrophically growing cells (Figure 2). Predictions indicate that acetate taken up into the cytoplasm was converted to acetyl-CoA by the enzyme acetyl-CoA synthase (ACS) in a single step. Acetyl-CoA then feeds into the TCA cycle, resulting in the conversion of oxaloacetate into citrate. A little over half of the citrate was converted into isocitrate; the remainder exported in exchange of malate. From isocitrate, most flux is predicted to be via isocitrate lyase (ICL), resulting in a “half TCA cycle” being operational, closely resembling the glyoxylate cycle. ICL ensures that the CO_2_ evolution steps of the TCA cycle are bypassed, resulting in retention of organic carbon by the cell. Model simulations are consistent with published experimental data, which have shown that ICL activity is essential for efficient mixotrophic growth (Plancke et al., 2014). After ICL, mixotrophic growth results in flux through succinate dehydrogenase (SUCDH(q8)m), producing reduced ubiquinol, associated with the mitochondrial electron transport chain feeding into oxidative phosphorylation. In addition to ubiquinol, the other product formed by SUCDH(q8)m is fumarate which is converted to malate by fumerase. Malate is seen to be shuttled in and out the mitochondrion, where export of malate in exchange for sodium ions is followed by a re-entry of malate in exchange for oxaloacetate. Oxaloacetate within the TCA was converted to phosphoenolpyruvate (PEP) the precursor compound of gluconeogenesis.

In phototrophic simulations, pyruvate, originating from carbon fixation is converted into acetyl-CoA and fed into the TCA cycle. ICL activity was redundant and the model chose to export isocitrate to the cytosol in exchange for malate. Cytosolic isocitrate was ultimately converted into biomass and the newly imported malate was converted into oxaloacetate and exported into the cytosol which fed back into pyruvate metabolism.

### Theoretical predictions suggest acetate increases glycolytic fluxes resulting in starch and sucrose accumulation

**Figure 3:**
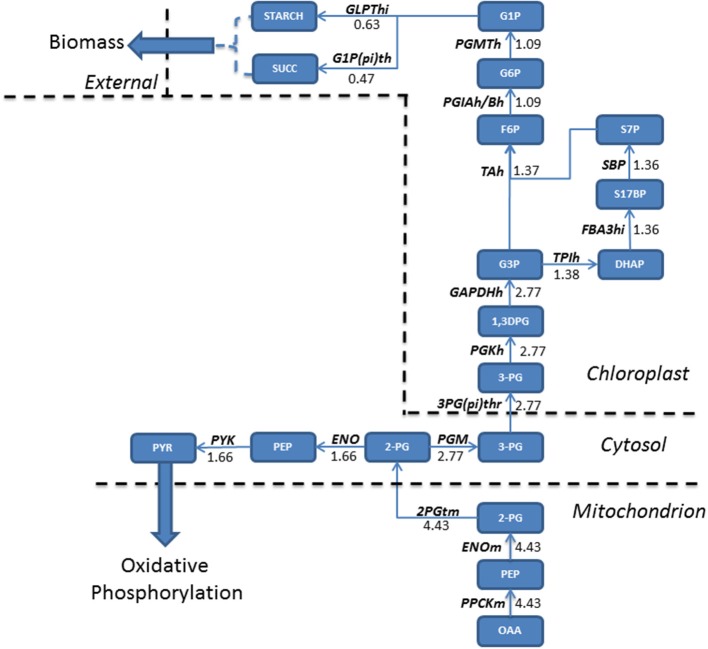
**Absolute flux (mmol gdw^−1^ h^−1^) residing within the glycolytic pathways were mapped for mixotrophic growth**. Oxaloacetate produced in the mitochondrion is the precursor molecule for gluconeogenesis. A relatively large flux entering gluconeogenesis allows for a branching of fluxes within the cytosol, with one branch feeding forwards into pyruvate metabolism, eventually fuelling oxidative phosphorylation, and the other continuing the gluconeogenetic pathway. As a result, both starch and sucrose accumulate within biomass when compared to phototrophically grown cultures. 1,3DPG, 3-Phospho-D-glyceroyl phosphate; 2-PG, Glycerate 2-phosphate; 2PGTm, Glycerate 2-phosphate transport; 3-PG, Glycerate 3-phosphate; 3PG(pi)thr, 3-Phospho glycerate/pi antiporter; DHAP, Dihydroxyacetone phosphate; ENO, Enolase; ENOm, Enolase (mitochondrial); F6P, Fructose 6-phosphate; FBA3hi, Sedoheptulose 1,7-bisphosphate D-glyceraldehyde-3-phosphate-lyase; G1P, Glucose 1-phosphate; G1P(pi)th, Glucose 1-phosphate/pi antiporter; G3P, Glyceraldehyde 3-phosphate; G6P, Glucose 6 phosphate; GLPThi, glucose-1-phosphate adenylyltransferase; OAA, Oxaloacetate; PEP, Phosphoenolpyruvate; PGIAh/Bh, Glucose-6-phosphate isomerase; PGKh, Phosphoglycerate kinase; PGM, phosphoglycerate mutase; PGMTh, phosphoglucomutase; PPCKm, Phosphophenol carboxykinase; PYK, pyruvate kinase; PYR, Pyruvate; S17BP, Sedoheptulose 1,7-bisphosphate; S7P, Sedoheptulose 7-phosphate; SBP, Sedoheptulose-bisphosphatase; SUCC, Succinate; TAh, Transaldolase; TPIh, Triosephosphate Isomerase.

In addition to increasing fluxes through enzymes involved in the TCA cycle and localized in the mitochondria, acetate also increased fluxes associated with the glycolytic pathway. Oxaloacetate, generated in TCA cycle reactions is converted into glycerate 2-phosphate (2PG) and exported into the cytosol, where a flux partition occurs, resulting in the majority of flux continuing through gluconeogenesis leading to the formation of both starch and sucrose, accumulating within biomass. Both starch and sucrose are the main products of photosynthesis (Klein et al., 1983). For higher plants sucrose is the main transport sugar but a lack of knowledge exists for the function of sucrose in algae. Flux not channeled into the gluconeogenesis is diverted toward the production of pyruvate within the cytosol, which is transported into the mitochondrion (Figure 3).

**Figure 4:**
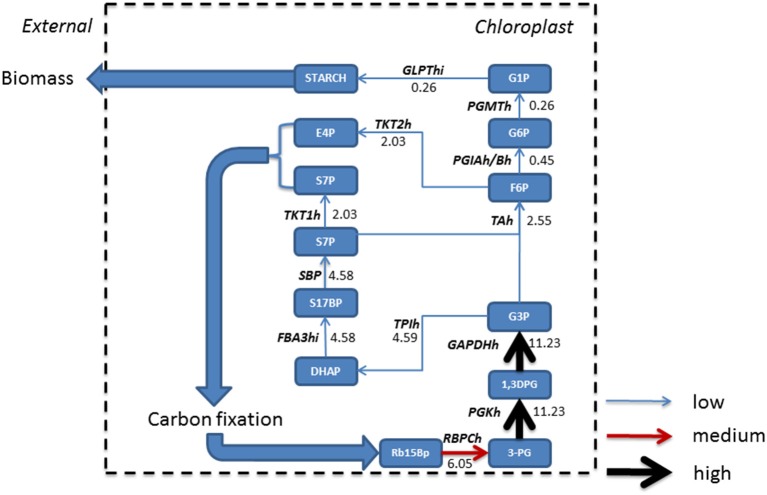
**Absolute fluxes (mmol gdw^−1^ h^−1^) residing within the glycolytic pathways were mapped for phototrophic growth**. Fluxes were categorized as low (<5 mmol gdw^−1^ h^−1^, thin blue arrow), medium, (>5 – 10 mmol gdw^−1^ h^−1^, red arrow), and high (>10 mmol gdw^−1^ h^−1^, thickest black arrows). Here, in the absence of acetate, gluconeogenesis starts in the chloroplast following from the production of triose sugars from carbon fixation within the chloroplast which is otherwise inactive under mixotrophic growth. A large resulting flux of triose phosphate allows for branching, with one branch accounting for the synthesis of pyruvate, and the other maintaining gluconeogenesis; however a further branch at fructose 6 phosphate forces flux back into the Calvin-Benson cycle to maintain CO_2_ fixation. As a consequence, less flux is carried to glucose 1-phosphate and ultimately allowing for the formation of starch only. 1,3DPG, 3-Phospho-D-glyceroyl phosphate; 3-PG, Glycerate 3-phosphate; E4P, Erythrose 4-phosphate; F6P, Fructose 6-phosphate; FBA3hi, Sedoheptulose 1,7-bisphosphate D-glyceraldehyde-3-phosphate-lyase; G1P, Glucose 1-phosphate; G3P, Glyceraldehyde 3-phosphate; G6P, Glucose 6 phosphate; GAPDHh, glyceraldehyde 3-phosphate dehydrogenase; GLPThi, glucose-1-phosphate adenylyltransferase; PGIAh/Bh, Glucose-6-phosphate Isomerase; PGKh, Phosphoglycerate kinase; PGMTh, Phosphoglucomutase; Rb15Bp, Ribulose 1,5-bisphosphate; RBPCh, Ribulose-bisphosphate carboxylase; S17BP, Sedoheptulose 1,7-bisphosphate; S7P, Sedoheptulose 7-phosphate; SBP, Sedoheptulose-bisphosphatase; TAh, Transaldolase; TPIh, Triose-phosphate isomerase; TKT1h, transketolase 1; TKT2h, transketolase 2.

Phototrophic metabolism (Figure 4) on the other hand resulted in the gluconeogenesis pathway commencing from chloroplastic glycerate 3-phosphate (3PG) derived from conversion of ribulose 1, 5-bisphosphate (Rb15Bp) and CO_2_ by Rubisco (RBPCh), the primary reaction of photosynthetic carbon fixation. The reactions of gluconeogenesis in the mitochondrion are otherwise inactive. The predominant flux from 3PG was branched toward the Calvin-Benson cycle in the chloroplast. A further branching of flux toward erythrose 4-phosphate (E-4P) and sedoheptulose 7-phosphate (S7P) and their immediate removal from gluconeogenesis toward carbon fixation is observed. The resulting flux toward glucose 1-phosphate (G1P) production was reduced due to branching in the network and when compared to mixotrophic simulations, was reduced by a factor of 2.4, and as such, only starch was accumulated within biomass.

### FBA predicts a suppression of photosynthetic CO_2_ fixation with acetate metabolism

**Figure 5:**
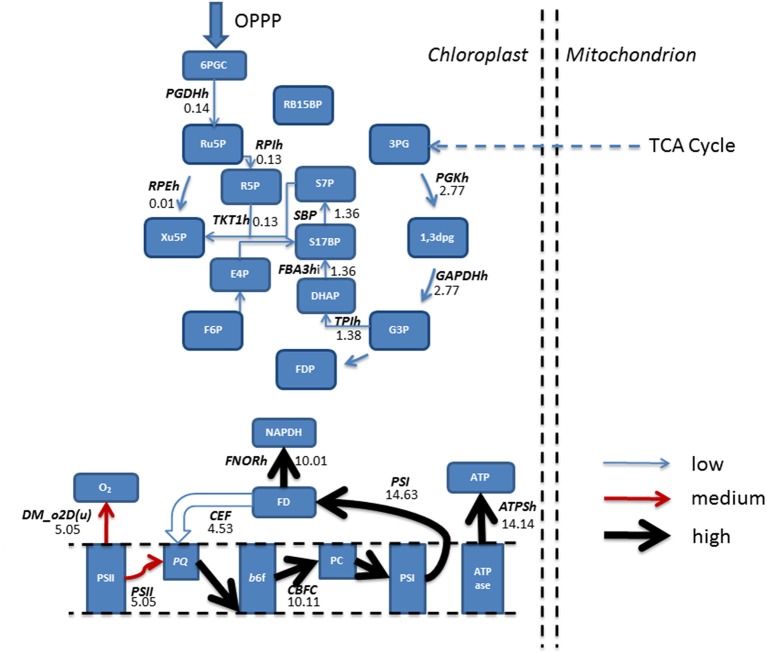
**Simulated predictions resulting from flux balance analysis were mapped onto reactions belonging to the Calvin-Benson cycle and photosynthetic reactions for mixotrophic growth**. Absolute fluxes (mmol gdw^−1^ h^−1^) were categorized as low (<5 mmol gdw^−1^ h^−1^, thin blue arrow), medium, (>5–10 mmol gdw^−1^ h^−1^, red arrow) and high (>10 mmol gdw^−1^ h^−1^, thickest black arrows). The simulations revealed an input of flux from the pentose phosphate pathway into the Calvin-Benson cycle, and flux being diverted away from CO_2_ and into ribulose 5-phosphate. Calvin-Benson cycle fluxes were further maintained by triose phosphate entry into the cycle, originating from within the mitochondrion. Upon inspection of photosynthetic fluxes, a cycling of electrons around photosystem I into the plastoquinone pool was active, resulting in flux diversion from the production of NAPDH back into the electron transport chain at plastoquinone. As a result of electron re-direction, ATP production is increased. 1,3DPG, 3-Phospho-D-glyceroyl phosphate; 3PG, 3-Phospho-D-glycerate; 6PGC, 6-Phospho-Gluconate; ATPase, ATP synthase; ATPSH, ATP producing synthase; b6f, Cytochrome *b*6F; CEF, Cyclic Electron Flow; DHAP, Dihydroxyacetone phosphate; DM_o2D(u), Oxygen Demand; E4P, Erythrose 4-phosphate; F6P, Fructose 6-phosphate; FBA3hi, Sedoheptulose 1,7-bisphosphate D-glyceraldehyde-3-phosphate-lyase; FD, Feredoxin; FDP, Fructose 1,6-bisphosphate; FNORh, Ferredoxin-NADP+ Reductase; G3P, Glyceraldehyde 3-phosphate; GAPDHh, Glyceraldehyde 3-phosphate dehydrogenase; OPPP, Oxidative Pentose Phosphate Pathway; PC, Plastocyanin; PGDHh, 6-Phosphogluconate Dehydrogenase; PGKh, Phosphoglycerate kinase; PQ, Plastoquinone; PSI, Photosystem I; PSII, Photosystem II; R5P, Ribose 5-phosphate; RB15BP, Ribulose 1,5-bisphosphate; RPEh, Ribulose-5-Phosphate; RPIh, Ribose-5-phosphate isomerase; Ru5P, Ribulose 5-phosphate, 3-Epimerase; S17BP, Sedoheptulose 1,7-bisphosphate; S7P, Sedoheptulose 7-phosphate; TKT1h, transketolase 1; TPIh, triose phosphate isomerase; Xu5P, Xylulose 5-phosphate.

In addition to altering fluxes in central carbon metabolism, acetate is also predicted to modulate fluxes in photosynthesis and the Calvin-Benson cycle. The Calvin-Benson cycle uses the products of photosynthetic electron transport, ATP and NADPH, to convert CO_2_ into organic compounds. In mixotrophic simulations (Figure 5) the Calvin-Benson cycle receives flux from the oxidative pentose phosphate pathway (OPPP) which is diverted away from CO_2_ fixation, but toward production of xylulose 5-phosphate (Xu5P) and fructose 6-phosphate (F6P) via the reversible action of the enzymes ribulose 5-phosphate epimerase (RPEh) and transketolase (TKT2h) respectively. It is with these reactions that the OPPP combines with the Calvin-Benson cycle and gluconeogenesis and ultimately demonstrates the interconnectivity of photosynthesis with central carbon metabolism.

Simulations in the presence of acetate predicted a decrease in oxygen evolution In line with experimental data (Heifetz et al., 2000), relative to the phototrophic state, as flux through PSII, the oxygen producing PS, decreased, and resulting in a reduced flux toward plastoquinone. In contrast, flux through PSI was increased, resulting from an increased flux through CEF, with flux back into cytochrome *b*_6_f from ferredoxin. As a result, ATP production resulting from CEF increased without production of NADPH, as described previously. The activity of CEF was otherwise non-evident with phototrophic simulations (Figure 5).

**Figure 6:**
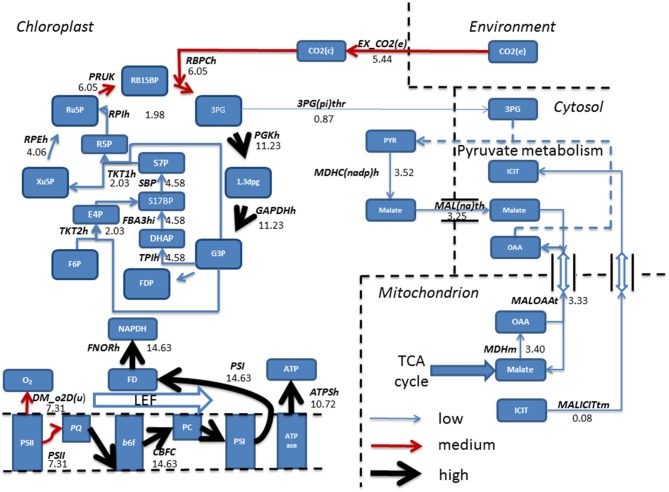
**Simulated fluxes (mmol gdw^−1^ h^−1^) resulting from flux balance analysis were mapped onto reactions belonging to the Calvin-Benson cycle and photosynthetic reactions for phototrophic metabolism**. Fluxes were categorized as low, (<5 mmol gdw^−1^ h^−1^, thin blue arrow), medium, (>5–10 mmol gdw^−1^ h^−1^, red arrow), and high (>10 mmol gdw^−1^ h^−1^, thickest black arrows). Simulations revealed fixation of CO_2_ into the Calvin-Benson cycle by the enzyme Rubisco allowing for the branching of flux at triose phosphate into pyruvate metabolism and ultimately entering the TCA cycle. Back in the chloroplast, a linear flow of electrons was observed resulting in production of NAPDH and ATP, providing the reducing power and energy to fuel the Calvin-Benson cycle. 1,3DPG, 3-Phospho-D-glyceroyl phosphate; 3PG, 3-Phospho-D-glycerate; 3PG(pi)thr, 3-Phospho glycerate/pi antiporter; ATPase, ATP synthase; ATPSH, ATP producing synthase; b6f, Cytochrome *b*6F; DHAP, Dihydroxyacetone phosphate; DM_o2D(u), Oxygen Demand; E4P, Erythrose 4-phosphate; EX_ CO_2_ (e), CO_2_ exchange; F6P, Fructose 6-phosphate; FBA3hi, Sedoheptulose 1,7-bisphosphate D-glyceraldehyde-3-phosphate-lyase; FD, Feredoxin; FDP, Fructose 1,6-bisphosphate; FNORh, Ferredoxin-NADP+ Reductase; G3P, Glyceraldehyde 3-phosphate; GAPDHh, Glyceraldehyde 3-phosphate dehydrogenase; ICIT, Isocitrate; LEF, Linear Electron Flow; MALICITtm, Malate/isocitrate antiporter; Mal(na)th, Malate transport; MALOAAt, malate/oxaloacetate antiporter; MDHC(nadp)hr, malate dehydrogenase; MDHm, malate dehydrogenase; OAA, Oxaloacetate; PC, Plastocyanin; PGKh, Phosphoglycerate kinase; PQ, Plastoquinone; PRUK, Phosphoribulokinase PSI, Photosystem I; PSII, Photosystem II; PYR, Pyruvate; R5P, RB15BP, Ribulose 1,5-bisphosphate; Ribose 5-phosphate; RBPCh, Ribulose-bisphosphate carboxylase; RPEh, Ribulose-5-Phosphate; RPIh, Ribose-5-phosphate isomerase; Ru5P, Ribulose 5-phosphate; S17BP, Sedoheptulose 1,7-bisphosphate; S7P, Sedoheptulose 7-phosphate; TKT1h, transketolase 1; TKT2h, transketolase 2; TPIh, triose phosphate isomerase; Xu5P, Xylulose 5-phosphate. Double arrows shown are representable of antiport reactions.

Steady state phototrophic conditions involved a linear flow of electrons through the photosynthetic reactions, resulting in large fluxes of oxygen evolution, NAPDH and ATP as electrons are passed along the photosynthetic chain. In addition, CO_2_ uptake into the chloroplast, and its fixation into the Calvin-Benson cycle carried predominant flux. Following this, the cycle allows for regeneration of Ru5P and RB15BP permitting fixation of another molecule of CO_2_. 3PG diverted away from the Calvin-Benson cycle was converted into pyruvate then malate. Malate entered the cytosol and transported into the mitochondrion in exchange for both oxaloacetate and isocitrate residing within the cytosol (Figure 6).

### Flux variability analysis determines essential reactions for phototrophic and mixotrophic growth

We have used FBA to investigate how acetate interacts with the central carbon metabolism on the global scale and in particular, the reaction of photosynthesis. By constraining the biomass reaction to the FBA predicted optima for each growth condition, the entire range of feasible minimal and maximal fluxes through each of the reactions were calculated using FVA. A reaction essentiality analysis combined the obtained fluxes resulting from FBA and FVA and identified reactions as being either essential, substitutable or blocked for phototrophic and mixotrophic growth (Table 1). We were keen to further investigate how essential the highlighted reactions from the selected subsystems that FBA predicted were to carry the predominant flux.

**Table 1:** **Reaction essentiality analysis for phototrophic and mixotrophic growth**.

**Reaction**	**Name**	**Equation**	**Phototrophic status**	**Mixotrophic status**
EX_ac(e)	Acetate exchange	[e]: ac <==>	Blocked	Essential
CSm	Citrate synthase, mitochondrial	[m]: accoa + h2o + oaa –> cit + coa + h	Substitutable	Essential
PPCKm	Phosphoenolpyruvate carboxykinase, mitochondria	[m]: atp + oaa –> adp + co2 + pep	Substitutable	Essential
RPEh	D-Ribulose-5-Phosphate 3-Epimerase	[h]: ru5p-D <==> xu5p-D	Essential	Substitutable
CO2t	CO_2_ transport, extracellular	co2[e] <==> co2[c]	Essential	Substitutable
ICL	Isocitrate Lyase	icit –> glx + succ	Substitutable	Essential
CO2tm	CO_2_ transport, mitochondrial	co2[c] <==> co2[m]	Essential	Substitutable
DM_photon(h)	Photon demand	[h]: photon450 –>	Essential	Essential
EX_photonVis(e)	Photon exchange	[e]: photonVis <==>	Essential	Essential
EX_o2(e)	O_2_ exchange	[e]: o2 <==>	Essential	Substitutable
DM_o2D(u)	Demand removing dummy O_2_ from system	[u]: o2D –>	Essential	Substitutable
O2t	O2 transport in via diffusion	o2[e] <==> o2[c]	Essential	Substitutable
PSII	Photosystem II	[u]: (2) h2o + (4) photon673 + (2) pq –> o2D + (2) pqh2	Essential	Substitutable
CBFC	Cytochrome b6/f complex	(2) h[h] + (2) pccu2p[u] + pqh2[u] –> (4) h[u] + (2) pccu1p[u] + pq[u]	Essential	Substitutable
PSI	Photosystem I	[u]: fdxox + (2) pccu1p + (2) photon680 + (2) h –> fdxrd + (2) pccu2p	Essential	Substitutable
FNORh	Ferredoxin-NADP+ reductase	fdxrd[u] + nadp[h] –> fdxox[u] + nadph[h] + h[h]	Essential	Substitutable

The vast majority of reactions for phototrophic and mixotrophic simulations were classified as flux substitutable [1549 reactions, (71%) and 1546 (70%) respectively]. Flux essential reactions for phototrophic growth and mixotrophic growth comprised the lowest subset of reactions with 16 essential reactions in each condition, representing less than 1% of reactions, whilst 626 reactions (28.6%) were blocked for phototrophic simulations verses 629 (28.7%) for mixotrophic growth.

Out of the 16 essential reactions identified under mixotrophic growth, 10 were also deemed essential for phototrophic growth. These reactions all consisted of photon exchange and uptake. The remaining six reactions were classified as substitutable (O2t, CO2tm, CSm, PPCKm) whilst acetate uptake (EX_ac(e)) and transport into the cytoplasm ACt were blocked. There were no blocked reactions observed for mixotrophic growth, however, there were seven substitutable reactions that were classed as essential for a phototrophic simulation. These included CO_2_ transport into the cytosol (CO2t), the conversion of ribulose 5-phosphate and Xu5P via ribulose-5-Phosphate 3-Epimerase (RPEh), cytochrome b6/f complex (CBFC), ferredoxin-NADP+ reductase (FNORh) and the two photosystems (PSI and PSII). Here, it seems that the activity of CSm, allowing for the entry of acetyl-CoA into the TCA cycle, and PEP carboxykinase suggest that TCA cycle itself is fundamentally important to provide a heightened biomass for mixotrophic growth, as these reactions were all substituble for phototrophic growth. The finding that CEF was substitutable for both growth regimes is important indicating its non-essential role in both mixotrophic and phototrophic metabolism.

### Cell physiology matches model simulations

We used the modified *i*CR1080 to predict primary metabolism with respect to phototrophic and mixotrophic growth. To further asses the reliability of model predictions, growth rate, and oxygen evolution, were measured to compare predicted growth and photosynthetic oxygen evolution to address the initial question of the reported down regulation of photosynthesis with mixotrophic growth.

**Figure 7:**
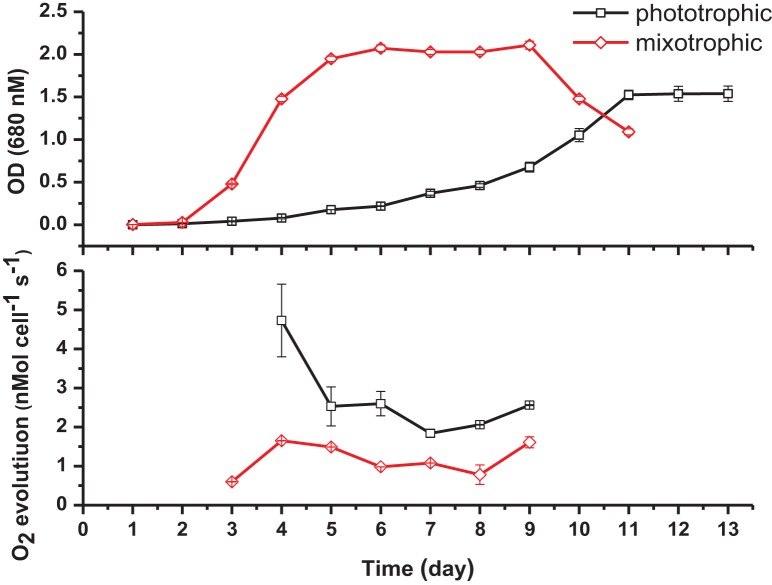
**Cell density measurements (top) and oxygen evolution as a measure of photosynthesis (bottom) under phototrophic and mixotrophic growth**. Despite supporting a higher growth capacity, acetate has the effect of decreasing oxygen evolution.

Specific cell growth rates under phototrophic and mixotrophic conditions were quantified for comparison with model predictions (Figure 7). *C. reinhardtii* cell growth occurred following a logistic curve for both phototrophic and mixotrophic cultures (Figure 7, top). Cell culture growth increased rapidly for mixotrophic cultures with cultures entering a stationary phase after 5–6 days. For phototrophically grown cells, stationary phase was not reached until day 11. Specific growth rates for phototrophic and mixotrophic cultures were calculated using data points obtained from a logarithmic phase of growth for both conditions. For mixotrophic cultures, the growth rate was calculated between days 3 and 4, and 9 to 11 for phototrophic cultures. Mixotrophic growth supported a growth rate 2.8 times greater than that of phototrophically grown cultures. FBA predicted growth rates in which acetate supported an *in silico* growth rate 2.38 times greater than phototrophic cultures, closely matching experimental data (Table 2).

**Table 2:** ***In silico* predictions and experimental data comparing phototrophic and mixotrophic specific growth and oxygen evolution rates**.

**Condition**	**Phototrophic growth rate**	**Mixotrophic growth rate**	**Fold change**
*i*CR1080 prediction (mmol gDw h^−1^)	0.135	0.321	2.38
Experimental result (OD day^−1^)	0.406 ± 0.04	1.130 ± 0.02	2.79
**Condition**	**Phototrophic O_2_ evolution**	**Mixotrophic O_2_ evolution**	**Fold change**
*i*CR1080 prediction (mmol gDw h^−1^)	7.31	5.05	0.70
Experimental result (nmol cell^−1^ s^−1^)	2.51 ± 0.19	1.51 ± 0.04	0.60

*Acetate had the effect of increasing the growth rate of cultures by a factor of 2.79 and decreasing oxygen evolution with a factor of 0.53 when compared to phototrophically grown cells. These factors are close to those predicted by the model. Experimental results were derived from triplicate biological replications. Error measurements represent that of the observed standard error*.

It has been widely documented that, despite promoting an enhanced growth rate, acetate results in inhibition of photosynthesis. We were able to quantify this effect and to compare results with the predictions of the modified version of *i*CR1080. The capacity for photosynthetic O_2_ evolution was estimated on a per-cell basis for both conditions during the exponential phase of growth, at time points when the cell density was sufficient to give measureable photosynthetic rates (Figure 7, bottom). *C. reinhardtii* grown under mixotrophic conditions exhibited a reduced capacity for photosynthesis, consistent with reported findings (Roach et al., 2013). The biggest comparable reduction in O_2_ evolution was observed at 4 days of growth. The oxygen evolution capacity of cultures was higher in phototrophic cultures than in mixotrophic on all days except at the final measuring day. Average oxygen evolution for phototrophic cultures was measured as 2.5 × 10^−7^ mMol O_2_ cell^−1^ s^−1^ whilst cells grown mixotrophically had an average capacity of 1.5 × 10^−7^ mMol O_2_ cell^−1^ s^−1^, a 0.6 fold decrease as a result of acetate addition. For simulations of phototrophic growth, a flux of 7.31 mMol gDw h^−1^ was estimated through the oxygen evolving reactions. Under mixotrophic constrains, a flux of 5.05 mMol gDw h^−1^ was associated with the oxygen evolution reaction of photosynthesis representing a 0.70 fold decrease (Table 2).

We thus conclude that our model, revised from the original *i*CR1080 model, is able to provide a realistic simulation of the responses of *C. reinhardtii* to acetate to show correlative trends between acetate metabolism, cell growth and oxygen evolution as markers of model validation. This model is able to predict and explain emergent physiological and metabolic changes that occur as a result of acetate addition.

## Discussion

It is widely accepted that growth in the presence of acetate induces a down regulation of photosynthesis in the model organism *C. reinhardtii* (Johnson et al., 2014). Here, we used a GEM (*i*CR1080) to evaluate the down regulation of photosynthesis associated with a mixotrophic growth regime, in the presence of acetate. Furthermore, model predictions have been validated using experimental results presented here and can be compared to results in the literature (Merchant et al., 2007; Roach et al., 2013). To the best of our knowledge, this is the first time this phenomenon of photosynthetic down-regulation in response to acetate has been addressed using predictive modeling. We furthermore combined this with a reaction essentiality analysis to gain more detailed understanding of the importance of specific reactions in this process.

Mixotrophic simulations included the uptake of acetate set at an experimentally determined level and allowed for the free exchange of gasses. It has been previously reported that the incorporation of acetate into acetyl CoA might occur via two possible pathways. The first pathway involves a two-step reaction involving ACK, prior to phosphate-acetyltransferase whilst the second pathway involves a single step conversion catalyzed by ACS (Johnson and Alric, 2013). Furthermore, acetate is thought to enter one of two cycles, either the glyoxylate or TCA cycle (Heifetz et al., 2000; Johnson and Alric, 2013). Boyle and Morgan (Boyle and Morgan, 2009), using a FBA model with 484 reactions which partially represented the compartmentalization of the cell and specifically did not take into account the presence of a glyoxylate cycle, predicted acetate entry into a complete TCA cycle under a heterotrophic growth condition. In the light however, they suggested a switch toward a modified TCA cycle using the enzyme ICL.

Different models contain different structures, constraints and different optimization criteria, hence used to address different questions. Based on the annotated genome and metabolic reconstruction, *C. reinhardtii* has the capacity to convert acetate to acetyl-CoA in the cytosol, into the mitochondria through the TCA cycle, into the glyoxylate cycle and in the chloroplast due to localization of the enzyme ACS in these organelles. Our analysis suggests that acetate taken up into the cytoplasm is converted to acetyl-CoA by the enzyme ACS. However, our essentiality analysis did show this reaction to be substitutable for both conditions. Therefore, we are not able to conclusively say which reaction is responsible for acetate assimilation.

Regardless of the primary acetate assimilatory reaction, we predict that acetyl-CoA feeds directly into a simplified TCA cycle, using the mitochondrial ICL with reducing power being consumed through oxidative phosphorylation. Operation of mitochondrial ICL was deemed essential for mixotrophic growth, but substitutable for phototrophic growth. Our model simulations are thus consistent with the experimental observation that acetate induces the expression of ICL and that ICL is required for growth on acetate (Plancke et al., 2014).

Acetyl-CoA was then fed into the mitochondria with the export of succinate toward mitochondrial respiration and increasing flux toward ubiquinol. In addition, acetate increased fluxes along the pathway of gluconeogenesis whilst suppressing CO_2_ fixation within the chloroplast, yet still exhibiting a greater biomass than phototrophically growing cells. The suppression of CO_2_ fixation by acetate has been predicted under a mixotrophic growth regime at low light (Boyle and Morgan, 2009). With regards to carbon fixation, *C. reinhardtii* mutants devoid of active Rubisco are still able to grow in the presence of acetate, albeit sub-optimally, compared to wild type (Pinto et al., 2013). Quantitative experimental evidence exists to describe that acetate addition decreases both carbon fixation and oxygen evolution by up to 50% (Heifetz et al., 2000). We have observed a similar trend in oxygen evolution as our model was predicting a photosynthetic decrease of 40% in mixotrophic conditions (Figure 5), however the model predicted a complete inhibition of CO_2_ fixation indicating an overestimation of the inhibitory effect of acetate.

We observe an increased flux through the CEF reactions, consistent with the suggestion that CEF around PSI explains the down-regulation of photosynthesis (Johnson and Alric, 2012). CEF involves the transfer of electrons from photosystem I to the plastoquinone pool via unknown electron carriers, then back to photosystem I via the cytochrome *b_6_f* complex. Previously, De Oliveira Dal'Molin et al. (2011) reported that CEF suppresses H2 production and suggested that hydrogen production and CEF are competing valves for the prevention of excess reducing equivalents (De Oliveira Dal'Molin et al., 2011). Consistent with this, our results indicate that CEF is the primary reaction rebalancing ATP and NADPH under mixotrophic conditions and that enhancing CEF optimizes biomass production. At least two distinct pathways have been proposed to account for CEF, being the PGR5 and NDH dependent pathways (Johnson, 2011). The general purpose of CEF is generally understood to be that of a protection against photo damage of PSI at high light intensities (Johnson et al., 2014). The way in which CEF elicits this protective role is generally accepted to be by sustaining an increased pH gradient across the thylakoid lumen, to regulate photon absorption (Johnson, 2011). CEF also functions to regulate the balance of ATP/NAPDH production resulting from the photosynthetic reactions. ATP can be regenerated when NADPH (at high concentrations) is consumed in an NDH cyclic electric flow, in the absence of linear electric flow, promoting proton translocation and generating a trans-thylakoid pH gradient (Peng et al., 2009). However, regarding algae, the formation of tightly associated super complexes involving PSI, cytochrome *b_6_f* complex and feredoxin NADPH oxidoreductase has been characterized (Iwai et al., 2010). It is believed that switching between cyclic and non-cyclic pathways provides a degree of flexibility in ATP and NAPDH production and hence plants and algae can adjust the ATP:NAPDH ratio to meet the demands of metabolism (Foyer et al., 2012). One hypothesis explaining how acetate decreases photosynthesis can be presented. Acetate provides an alternative reduced source of carbon to CO_2_. As such, our mixotrophic simulations suggest *C. reinhardtii* prefers to assimilate acetate rather than CO_2_ by way of suppressing the CO_2_ fixation step, thereby alleviating the requirement for NADPH. Acetate assimilation via ACS however still requires ATP (Morales-Sanchez et al., 2015). The ratio for ATP: NAPDH would therefore change resulting from acetate metabolism. To facilitate this change, a shift in electron flow form a linear to a cyclic flow is one way in which the cell can meet this demand. As explained previously, CEF results in an injection of electrons to circumnavigate the oxygen evolving photosystem of photosynthesis, explaining a decrease in oxygen evolution. The non-essentiality of CEF as suggested by the model has been supported experimentally as mutants devoid of PGR mediated CEF still grow, however sub-optimally (Johnson et al., 2014).

Here we have presented a GEM which is able to predict and explain the role of CEF in acetate metabolism while demonstrating the non-essentiality of CEF. It is well known that acetate metabolism leads to an increased growth rate and biomass. Model simulations are able to reproduce this and suggest that an enhanced biomass is achieved in mixotrophic cells by increased fluxes into starch and sucrose metabolism resulting from increased gluconeogenesis. In addition, the OPPP was active resulting in the incorporation of 6PGC into R5P. R5P is used in the synthesis of nucleotides and nucleic acids, supporting greater cell profilation and increased biomass (Bar-Peled and O'Neill, 2011). Despite the fact that acetate metabolism and photosynthesis are deeply connected, the idea of a complete inhibition of carbon fixation does however remain too simplistic.

As a tool, flux balance models are becoming increasingly popular for explaining biological phenomena. As we have shown here, FBA is a powerful tool for data qualification. Of course, assumptions are made in constraint based modeling approaches and, as such, the questions investigated need to be applicable to the methodology being used. To accurately predict the fate of acetate using constraint based modeling techniques presents a challenge due to the presence of isozymes. To fully constrain the solution space requires integration of targeted omics data.

We have modeled acetate metabolism in the light and as such, in a photosynthetic organism, metabolic pathways that are specifically active in the night are ignored. For example, starch degradation was constrained to zero to reflect the suppression of this reaction in the light. As a result, although starch contributed to the biomass, its accumulation was not specifically optimized account for low synthesis of starch. Experimental studies have shown that phototrophic growth favors starch accumulation when compared to mixotrophic cultures (Singh et al., 2014). This is a problem that it may in the future be possible to address by using advanced techniques of flux modeling such as dynamic FBA.

Just as the ratio of ATP and NADPH have been shown to modulate the reactions of photosynthesis, elemental ratios of carbon and nitrogen have also been shown to affect cellular metabolism of photosynthetic microalgae (Talmy et al., 2014). Nitrogen and carbon rich molecules such as amino acids are stoichiometry constrained with the biomass reaction. As previously described, there was an overall increase in biomass production with acetate metabolism; however there was no evidence for a specific up regulation of reactions involved with nitrogen metabolism. Nitrogen assimilation reactions increased in response to acetate in line with increase in biomass. This reflects one limitation of the modeling approach used which might be addressed using an elemental analysis of cells grown in both conditions to adjust the biomass composition for each case.

In conclusion, we have produced a curated version of a GEM of *Chlamydomonas reinhardtii*, with an accurate representation of CEF, which is now able to explain the down regulation of photosynthesis observed under mixotrophic growth conditions in the presence of acetate. The predictive capabilities of the model were used to highlight changes in metabolism that were essential for this trait. We can conclude that acetate incorporation into metabolism is achieved via reactions of the TCA cycle before entering central metabolism requiring CEF. CEF serves to support the production of a proton motive force that allows ATP generation without the net formation of NADPH, serving as a balance to meet metabolic demands imposed on the system. The functioning of CEF is however non-essential. In doing so, we have demonstrated how the reactions of photosynthesis interconnect with carbon metabolism on a global scale, and how systems approaches play a viable tool in understanding complex relationships at the cell-wide scale.

## Conflict of interest statement

The authors declare that the research was conducted in the absence of any commercial or financial relationships that could be construed as a potential conflict of interest.
