# Corrigendum: Flux balance analysis reveals acetate metabolism modulates cyclic electron flow and alternative glycolytic pathways in *Chlamydomonas reinhardtii*

**DOI:** 10.3389/fpls.2016.00362

**Published:** 2016-03-22

**Authors:** Stephen P. Chapman, Caroline M. Paget, Giles N. Johnson, Jean-Marc Schwartz

**Affiliations:** Faculty of Life Sciences, University of ManchesterManchester, UK

**Keywords:** glycolysis, mixotrophic growth, metabolic model, photosynthesis, green algae, flux balance analysis, acetate metabolism, cyclic electron flow

Reason for Corrigendum:

There was a mistake with the naming of the genome-scale metabolic model of *Chlamydomonas reinhardtii* originally published in Molecular Systems Biology in 2011 (Chang et al., 2011). In our article there are 10 instances where we refer to the model as *i*CR1080, however the correct name should be *i*RC1080, named after the initials of the researcher who reconstructed it. The authors apologize for the mistake. This error does not affect the scientific conclusions of the article in any way.

## Author contributions

All authors listed have made substantial, direct and intellectual contribution to the work, and approved it for publication.

### Conflict of interest statement

The authors declare that the research was conducted in the absence of any commercial or financial relationships that could be construed as a potential conflict of interest.
